# Carbon Dioxide Inhalation Induces Dose-Dependent and Age-Related Negative Affectivity

**DOI:** 10.1371/journal.pone.0000987

**Published:** 2007-10-03

**Authors:** Eric J. Griez, Alessandro Colasanti, Rob van Diest, Ewa Salamon, Koen Schruers

**Affiliations:** 1 Department of Psychiatry and Neuropsychology, University of Maastricht, Maastricht, The Netherlands; 2 Department of Psychiatry and Behavioural Neuroscience, Upstate Medical University, State University of New York, Syracuse, New York, United States of America; University of Sydney, Australia

## Abstract

**Background:**

Carbon dioxide inhalation is known to induce an emotion similar to spontaneous panic in Panic Disorder patients. The affective response to carbon dioxide in healthy subjects was not clearly characterized yet.

**Methodology/Principal Findings:**

Sixty-four healthy subjects underwent a double inhalation of four mixtures containing respectively 0, 9, 17.5 and 35% CO_2_ in compressed air, following a double blind, cross-over, randomized design. Affective responses were assessed according to DSM IV criteria for panic, using an Electronic Visual Analogue Scale and the Panic Symptom List. It was demonstrated that carbon dioxide challenges induced a dose dependent negative affect (p<0.0001). This affect was semantically identical to the DSM IV definition of panic. Older individuals were subjectively less sensitive to Carbon Dioxide (p<0.05).

**Conclusions/Significance:**

CO_2_ induced affectivity may lay on a continuum with pathological panic attacks. Consistent with earlier suggestions that panic is a false biological alarm, the affective response to CO_2_ may be part of a protective system triggered by suffocation and acute metabolic distress.

## Introduction

Intolerance to carbon dioxide in anxiety prone individuals has been widely documented [1]–[3]. When inhaling hypercapnic gasses, subjects diagnosed with Panic Attacks (PA) shortly sense an instant affect that closely replicates spontaneous panic [4]. Hence Klein inferred that pathological PA's may be false biological alarms, resulting from neuronal misfiring in an evolutionarily evolved, CO_2_ driven oversensitive suffocation monitor [5].

In other words it was suggested that panic may be an inborn behavioural response to a metabolic distress. If so, panic must belong to the behavioural repertoire of healthy individuals, the hypersensitive alarm in PD subjects corresponding to a normoresponsive system in others. Accordingly, the very same mechanism firing false alarms in PD patients as a response to moderate CO_2_ intake, should be activated in healthy subjects following higher doses of CO_2_.

Here we demonstrate in healthy individuals that increasing concentrations of CO_2_ dose dependently induce a negative affect and that this affect is semantically identical to panic, as defined in current psychiatric nosology.

## Materials and Methods

### Subjects

Sixty-four volunteers provided their informed consent to participate in the study. There were 33 males and 31 females, aged 35.8 (SD = 15.9) and 31.1 (SD = 14.4) years respectively.

All potential participants had a complete inventory of medical history and a physical examination. Inclusion criteria were 18 to 65 years of age and a good present and past physical and mental condition. The mental condition was assessed by a structured psychiatric interview (Mini International Neuropsychiatric Interview) performed by a psychologist who was not directly involved in the study. Exclusion criteria included a history of pulmonary or cardiovascular disease, the presence of hypertension (diastolic>100 mmHg; systolic>170 mmHg), cerebral aneurysm, pregnancy, epilepsy, excessive smoking (>15 cigarettes/day), use of adrenergic receptor blockers and use of psychotropic medication. A history of affective or anxiety disorders within a first-degree relative excluded participation. Participants were also excluded if they reported common specific fears or if there was any suspicion of history of Panic Attacks. The ethics committee of the Academic Hospital of Maastricht approved the study.

### Procedure

The inhalation apparatus and the general procedures used in our laboratory have been described elsewhere [6].

More specifically, the procedure consisted for each subject in a double inhalation of four mixtures containing respectively 0, 9, 17.5 and 35% CO_2_ in compressed air, following a double blind randomized design.

Subjects were instructed in the use of a mask with a demand valve for self-administration of medical gasses and told that they would take a double vital capacity breath of four different concentrations of CO_2_ in air, which, though being a harmless physiologic substance, may cause brief neurovegetative responses and arousal or anxiety, depending on the concentration. Subjects were asked to exhale to the maximum, to position the inhalation mask on their face and inhale their full capacity as quickly as possible. Next they were to empty their lungs and refill them immediately with gas, whereupon they had to hold their breath for 5 seconds before exhaling. All inhalations took place within one week, on four separate days but at the same time for each probant. Care was taken that each inhalation represented at least 80% of the subject's vital capacity.

### Assessments

Affective responses were assessed with strict reference to the DSM IV (APA, 2000 [7]), which refers to a PA as “a discrete period of intense fear or discomfort”, in which four (or more) out of a list of thirteen predefined symptoms develop abruptly and reach a peak within 10 minutes.

Accordingly (Fig. S1) we used an electronic visual analogue scale for affect (eVAAS). The eVAAS was programmed on a Compaq Tablet PC, TC1000, with a 21,0 cm×16,0 cm touch screen having a 1027×748 pixel resolution. The VAAS was a 20 cm×1 cm horizontal bar. Subjects had to mark their anxiety level by tipping on the bar with a stylus, which had a 1 mm diameter spherical tip. The top of the display was labelled “*fear or discomfort*”. The scale was anchored from 0, “no fear/discomfort at all”, to 100, “the worst imaginable fear/discomfort ”. This instrument has been validated for use during 35% CO_2 _challenges [Van Duinen, M.A., Rickelt, J., Griez, E.J.L.. Validation of the eVAAS. 2007 unpublished].

Panic Symptom List (PSL-IV) was used to evaluate panic symptomatology [8]. It consisted in a questionnaire listing thirteen items, each item representing a DSM-IV panic symptom, to be rated on a five point scale, from 0 (absent) to 4 (very intense) (Fig. S1).

The eVAAS was presented at baseline immediately before inhalation, which was followed after CO_2_ by multiple instant assessments, in fact as many as possible, during 60 seconds. This allowed the computation of both a peak value and an area under the curve (AUC). The Panic Symptom List was administered one minute before and after each inhalation. The total PSL score was calculated for each assessment.

### Statistical analysis

Statistical analysis was performed on the eVAAS peak values, obtained by subtracting the baseline from the maximum value, and PSL total scores, represented by delta scores (post-pre assessment).

A one-way Manova of repeated measures with eVAAS peak values as the dependent variable and dose (exposure to the four mixtures of CO_2_) as the within-subjects factor was used to investigate the affective response to the various CO_2_ mixtures. The same analysis was conducted with eVAAS AUC scores and PSL individual and total scores as the dependent variables.

AUC was calculated by the trapezoidal rule extrapolation method. The data were reanalysed using a repeated measures design with eVAAS peak values or PSL value as the dependent variable, age (below and above age 38) as a between-subjects factor and exposure to the four mixtures as the within-subjects factor. In both analyses, orthogonal polynomial trend contrasts were used to search for the presence of significant linear and/or quadratic trends in case of a significant “dose” effect.

Subjects were divided in “responders” and “non-responders”, according to conservative criteria, proposed by others in previous CO_2 _challenge studies [9]. Following those criteria an arbitrary eVAAS peak score of 50 was used as threshold to identify the responders (mean of the eVAAS peak scored during AIR+2 SD). In addition responders should report at least one-point increase for at least four of the 13 PSL symptoms.

## Results

**Figure 1:**
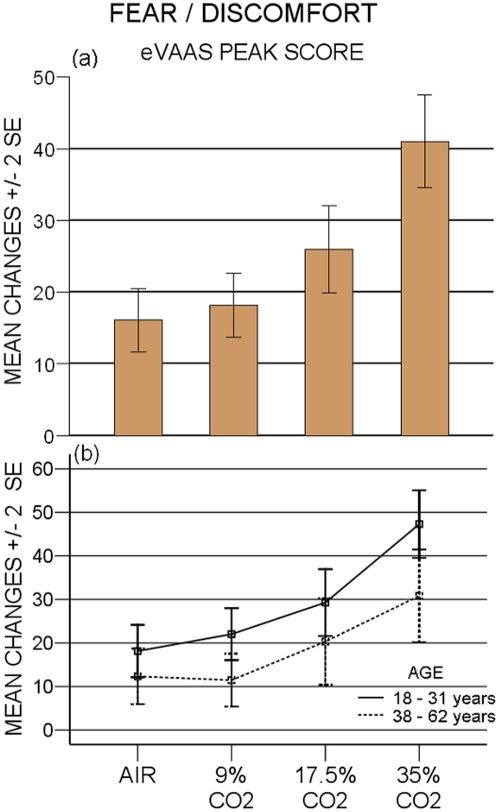
Peak scores on the Fear/Discomfort scale in four different CO_2 _conditions. a) eVAAS: air vs 9% p = 0.44; air vs 17.5% and vs 35% p ≤ 0.001; 9% vs 17.5% and vs 35% p ≤ 0.0001; 17.5% vs 35% p≤0.0001. b) Younger versus older subjects: p<0.05

Results are presented in figures 1–5.

**Figure 2 pone-0000987-g002:**
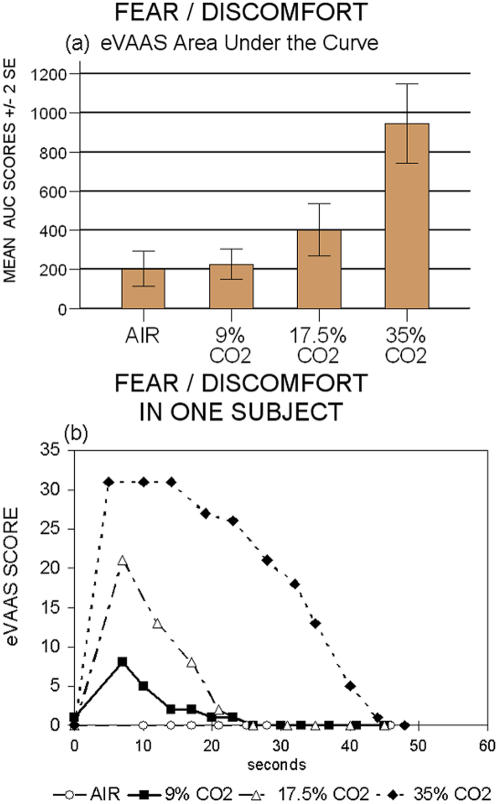
Area Under the Curve on the Fear/Discomfort scale in four different CO_2 _conditions. a) eVAAS AUC score: air vs 9% p = 0.91; air vs 17.5% and vs 35% p ≤ 0.005; 9% vs 17.5% and vs 35% p ≤ 0.001; 17.5% vs 35% p≤0.0001. b) Time course of Fear/Discomfort in a single subject after the double inhalation of 0%, 9%, 17.5%, 35% CO_2_ respectively.

**Figure 3 pone-0000987-g003:**
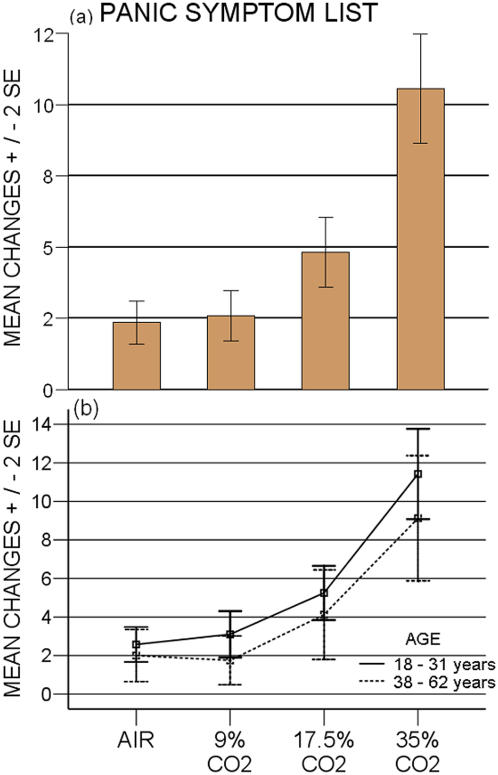
Intensity of PSL panic symptoms in four different CO_2 _conditions. a) PSL: air vs 9% p = 0.76; air vs 17.5% and vs 35% p ≤ 0.0001; 9% vs 17.5% and vs 35% p ≤ 0.0001; 17.5% vs 35% p ≤ 0.0001. b) Younger versus older subjects: p = 0.217

**Figure 4 pone-0000987-g004:**
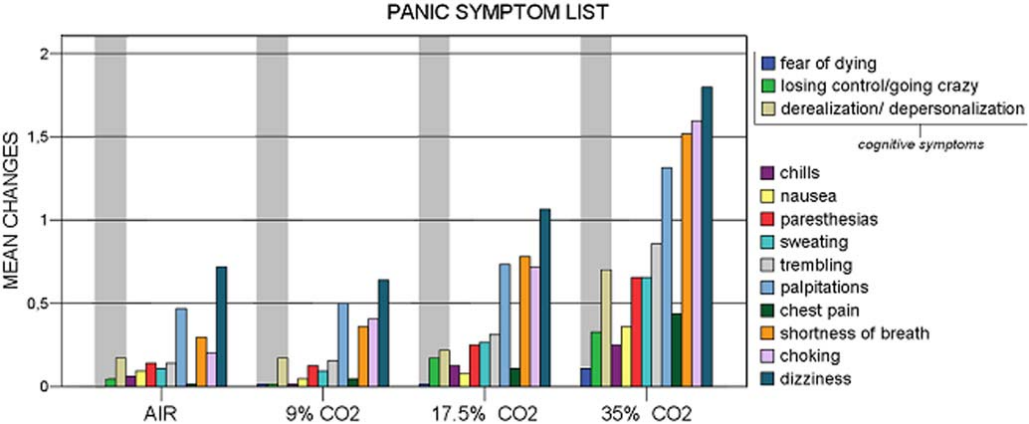
DSM panic symptoms intensity in healthy subjects taking four different doses of CO_2_.

**Figure 5:**
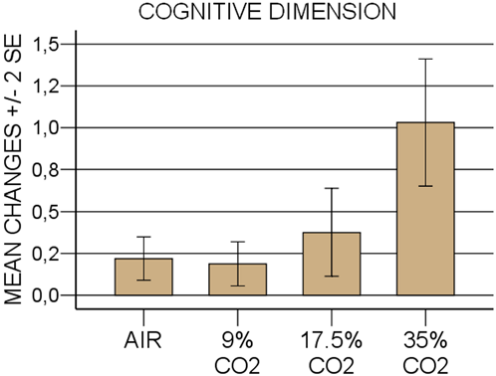
Aggregate score of cognitive symptoms induced by four different CO_2 _conditions in healthy subjects (“derealization-depersonalization” score+ “fear of loosing control-going crazy” score).

eVAAS peak values showed CO_2_-triggered affectivity to be dose dependent (p<0.001), displaying an increase with concentration, which fits both a significant linear (p<0.0001) and quadratic pattern (p<0.001) (Fig. 1 a). eVAAS AUC values were also dose related (p<0.0001) (Fig. 2 a,b) and exhibited a significant linear and quadratic pattern (p<0.0001). PSL data yielded similar results, with comparable dose-response relationship (p<0.0001) and mathematical pattern (p<0.0001) (Fig. 3 a).

Changes in individual PSL scores symptoms are presented in Fig. 4. As far as cognitive symptoms are concerned, a PSL score increase >1 in any of CO_2_ conditions was shown in 53% of the subjects.

The sum of the symptom scores, defining a cognitive dimension (“derealization-depersonalization” score+“fear of loosing control-going crazy” score) [10], [11] is presented in Fig. 5. Looking to the cognitive symptoms separately (to which we added fear of dying), 48%, 26%, and 8% of the subjects reported an increase of PSL score respectively, in any of CO_2_ condition. A significant CO_2_ dose-dependent relationship was evidenced for derealization-depersonalization, and fear of loosing control-going crazy (p<0.0001), which displayed a linear (p <0.001) and quadratic pattern (p<0.05). A similar significant dose-effect was found for fear of dying (p<0.05), however it appeared to be overall a very rare symptom.

According to above defined criteria there were 4 (6%), 4 (6%), 9 (14%), and 24 (37%) “responders” in the 0, 9, 17.5 and 35% CO_2_ conditions respectively (p≤0.0001).

Dividing the subjects in an older (> 38 years) and younger group, analysis revealed a significant difference in eVAAS peak scores (p<0.05) (Fig. 1 b). eVAAS peak values in both age groups increase in a significant linear (p<0.001) and quadratic pattern (p<0.001) and run parallel. PSL scores displayed that age effect was not significant (p = 0.217) (Fig. 3 b).

Dividing the subjects by gender, no significant differences were found between males and females in any of the assessed parameters (eVAAS peak score, eVAAS AUC score, PSL–IV score).

## Discussion

The double breath challenge induced an instant affect with a negative valence. As rated on the eVAAS, the healthy volunteers experienced a significant sense of “fear” or “discomfort” while reporting substantial panic symptomatology on the PSL. Both eVAAS and PSL were strictly based on DSM-IV semantics. The picture as a whole was a mathematical function of CO_2_ intake.

It may therefore be inferred that a double breath of increasing concentrations of CO_2_ dose dependently induced a condition complying with the formal criteria of panic in current psychiatric nosology.

There has been a wealth of evidence showing that experimental hypercapnia triggers PA's in patients diagnosed with PD [1], [2], [4], and conditions which are closely related to PD [12], [13]. In contrast, the same procedure failed to affect patients with other disorders [14], in particular those with Generalized Anxiety Disorder [15], [16], Obsessive-Compulsive Disorder [17], [18], Eating Disorders [19], Major Depression [20] and control groups of healthy volunteers. First-degree relatives of PD patients however share a significant degree of CO_2_ vulnerability [4], [21], [22]. In fact, the liability to experience panic with CO_2_ exposure discriminates between individuals at high and low risk for PD [3].

Recent reports have suggested that healthy individuals breathing a low 7% concentration of CO_2_ may display signs of generalized anxiety [9], [23]. Yet, the present results are the first to demonstrate that CO_2_ dose dependently activates a condition identical to panic in healthy volunteers, regardless of any constitutional predisposition to psychiatric pathology.

Did CO_2_ induce a true emotion? While formally meeting all the criteria of a PA according to modern psychiatric nosology, the CO_2_ induced state we observed in our healthy subjects may have been a “phenocopy” of panic, the amalgam of autonomic symptoms of hypercapnia and some resulting physical discomfort. Amongst the 13 PSL items, we therefore separately analyzed the specific cognitive symptoms of “derealization” and “fear of loosing control”. Several studies have identified these symptoms as belonging to a specific psychological/cognitive cluster on basis of factor analysis [10], [11]. Our results show that both “derealization” and “fear of loosing control” were linked to the doses of inhaled carbon dioxide in a significant linear and quadratic pattern. Across the procedure, “derealization/depersonalisation” displayed more than one point increase (on a five point scale) in about half of the subjects, and “fear of loosing control” in about one fourth of them. Fear of dying did not belong to the cognitive dimension in Meuret and Cox's studies [10], [11], nevertheless, from a conceptual point of view, it may refer to an extreme type of emotion. In the present study “fear of dying” remained very infrequent. However, when reported, we noted a significant dose-response relationship. It should be born in mind that all subjects were in a safe laboratory environment, and all had received ample reassurance regarding the safety of the intervention trough the informed consent procedure. This obviously influenced the psychological impact of CO_2_. Yet, modest as they are, cognitive shifts did occur, they were a function of the experimental procedure, and their occurrence was statistically significant.

This lends support to the idea that, beyond a particular threshold, carbon dioxide may yield genuine psychotropic properties in healthy individuals.

Influential authors have increasingly referred to emotions as evolutionarily derived, “organism-ready solutions” to face major survival problems [24], as brain representations of internal body states [25], and more specifically, as images of the “material me” arising from “the homeostatic condition of each individual's body” [26]. The idea that panic may proceed from a suffocation alarm disrupted by acute CO_2_ loading is perfectly consonant with such views. Several pieces of evidence point to a connection between hypercapnia and emotion. For instance, it appears that central chemosensitivity is not restricted to medullary respiratory neurons. Severson and co-workers [27] have shown that midbrain raphe serotonergic neurons are CO_2_ sensors, and midbrain neurons are not believed to have any direct function in the control of ventilation. Instead these midbrain chemosensors head mainly in the rostral direction. They have been proposed to participate in the homeostasis of the brain via non-respiratory responses to hypercapnia, including behavioural reactions as hyperarousal and anxiety [28]. Liotti et al. have produced neuroimaging evidence linking directly CO_2_ inhalation with brain structures related to emotions [29]. Following CO_2_ induced breathlessness, healthy volunteers displayed limbic and paralimbic activation, and neuronal firing in the affective brain correlated with the sense of suffocation. The authors comment that this neuronal activity may reflect a primal emotion, in other words “a compelling interoceptor-driven affect, rooted in metabolic distress, and aimed at signalling that the existence of the organism is endangered.” In an earlier study on the characteristics of CO_2_ induced responses, healthy volunteers spontaneously described their subjective experience as “frightening”, “panicky” or “scaring”, while authors noted that the sensitivity of the feeling, which was poorly correlated with the ventilatory response, varied threefold among individuals [30].

Our study shows that CO_2_ intake induces an affective state, which is similar to the psychiatric picture of panic. Within subjects, we observe a significant interaction between the intensity of the affective response and the CO_2_ concentration of the inhaled mixture.

We show older subjects to display less behavioural vulnerability to CO_2, _compared to younger individuals. To the extent that CO_2_ intake is a valid model of panic, this difference between younger and older subjects strikingly evokes the decline of natural PA's and the progressive blunting of panic symptomatology in PD patients when they grow older [31]. Most studies have found a lower prevalence of PD amongst elderly people [32]. The decreased CO_2_ susceptibility in the elderly revealed by the present data reminds of a similar age effect found with experimental cholecystokinin provocation of panic [33]. If midbrain serotonergic chemosensors are at work in the chain of events leading from CO_2_ to panic, the phenomenon observed in our study might be related to an age dependent decline of serotonergic activity [34], [35].

No gender differences were found. This is somewhat surprising in view of all epidemiological data showing women to be at greater risk for panic than men [36]. Yet, a recent study in a nonclinical population shows women reporting more fear and panic than men after CO_2_ administration [37]. Interestingly, when asked to rate their experience on a “like or dislike” dimension (which dimension has a conceptual overlap with “discomfort”), the gender difference disappeared. This suggests women being more prone than men to report a feeling as “anxiety”. Therefore, lumping together anxiety and discomfort in our eVAAS may have blunted a gender effect. It is noteworthy that the few existing reports about sex differences in the so-called condition “non fearful PD”, which diagnosis relies on “discomfort” rather than on “anxiety”, suggest that both genders have similar prevalence [38], [39].

A final comment applies to the potential of further work with CO_2_ challenges in healthy individuals. The panic model of CO_2_, in particular the single breath 35% CO_2_ procedure, has proven to be both valid and reliable [40]. It has undergone extended pharmacological validation, e.g. Bertani, Perna et al. [41]. Assuming that higher doses of CO_2_ activate the same physiologic chain of events in panic free individuals, CO_2_ challenges in healthy volunteers may have a strong potential as a substitute to early clinical trials in testing novel pharmacological compounds.

In conclusion, it appears that healthy individuals display a distinct behavioural vulnerability to increasing levels of acute hypercapnia. This effect is dose-dependent and shares a striking similarity with the psychiatric condition of panic.

CO_2_ susceptibility, sensed as acute affective distress may represent an evolutionarily evolved protective mechanism in case of impending asphyxia.

## Supporting Information

Figure S1 Experimental assessments. DSM IV TR criteria for Panic Attack; eVAAS for Fear/Discomfort; Panic Symptom List (PSL-IV)
(0.14 MB TIF)
