# Supplementary figures and images for: Carbon Dioxide Inhalation Induces Dose-Dependent and Age-Related Negative Affectivity
Source: PLoS One. 2007 Oct 3;2(10):e987. doi: 10.1371/journal.pone.0000987 (PMC1991589; doi:10.1371/journal.pone.0000987)

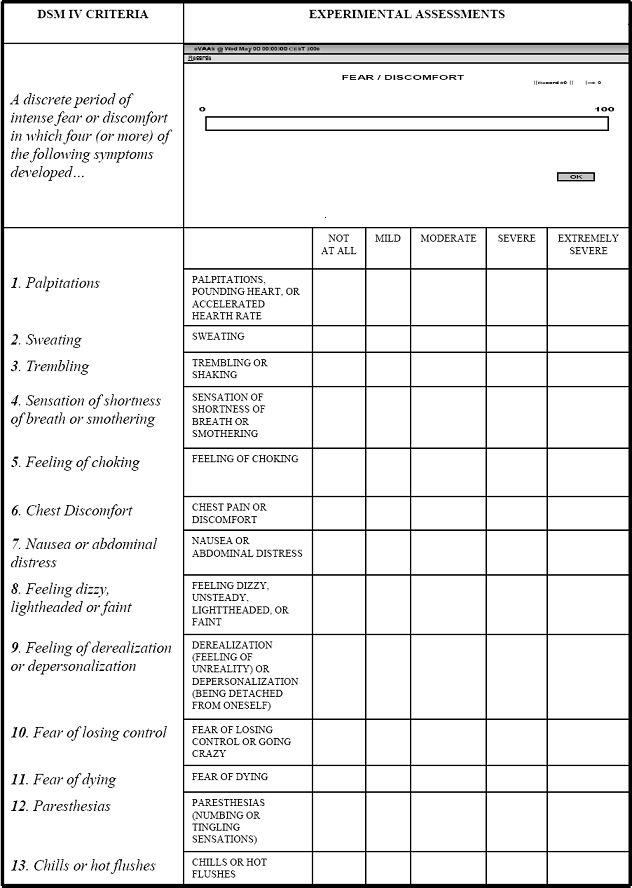

Supplement: Figure S1 — Experimental assessments. DSM IV TR criteria for Panic Attack; eVAAS for Fear/Discomfort; Panic Symptom List (PSL-IV) (0.14 MB TIF) [file pone.0000987.s001.tif]
